# Synergistic effects of natural compounds and conventional chemotherapeutic agents: recent insights for the development of cancer treatment strategies

**DOI:** 10.1016/j.heliyon.2022.e09519

**Published:** 2022-05-24

**Authors:** Ana María Castañeda, Carlos Mario Meléndez, Diego Uribe, Johanna Pedroza-Díaz

**Affiliations:** Grupo de Investigación e Innovación Biomédica GI^2^B, Facultad de Ciencias Exactas y Aplicadas, Instituto Tecnológico Metropolitano, 050012, Medellín, Colombia

**Keywords:** Chemotherapy, Natural products, Tumoricidal effect, Reversal of chemoresistance, Selectivity for cancer cells

## Abstract

Cancer is one of the leading causes of death in the world. Chemotherapy is presented as an option for treatment of this disease, however, low specificity, high resistance rates, toxicity and hypersensitivity reactions, make it necessary to search for therapeutic alternatives that increase the selectivity of treatment, reduce the side effects and enhance its antitumor potential. Natural products are accessible, inexpensive and less toxic sources; in addition, they have multiple mechanisms of action that can potentiate the outcome of chemotherapeutics. In this review, we present evidence on the beneficial effect of the interaction of dietary phytochemicals with chemotherapeutical agents for cancer treatment. This effect is generated by different mechanisms of action such as, increased tumoricidal effect via sensitization of cancer cells, reversing chemoresistance through inhibition of several targets involved in the development of drug resistance and, decreasing chemotherapy-induced toxicity in non-tumoral cells by the promotion of repair mechanisms. Studies discussed in this review will provide a solid basis for the exploration of the potential use of natural products in combination with chemotherapeutical agents, to overcome some of the difficulties that arise in the management of cancer patients.

## 1 Introduction

Cancer biology is complex and its understanding represents a challenge for the development of efficient therapies, in order to overcome the drawbacks of traditional chemotherapy (systemic toxicity, low specificity and high resistance rates) and which limit the clinical response. Fortunately, in the last decades remarkable progress into the mechanisms of cancer pathogenesis has been achieved, which has allowed to determine the “hallmark” capabilities of cancer cells during tumor onset and progression and also, the exploration of new cancer treatment strategies [1, 2, 3].

Recently, the administration of multiple chemotherapeutic drugs with different biochemical and molecular targets, known as combined chemotherapy, has enabled efficacy to be improved and adverse effects to be reduced [4, 5]. Moreover, the search for natural alternatives that present lower toxicity has taken on more importance in recent years. Experimental evidence suggest that the combination of antitumoral agents with different natural compounds, such as curcumin, resveratrol and epigallocatechin-3-gallate (EGCG), among others, have the potential to reduce cancer treatment resistance and to perform chemoprotective actions (Table 1) [6, 7, 8].

**Table 1:** Combinatorial effect of natural compounds with chemotherapy. Experimental evidence about combination of antitumoral agents with different natural compounds and their potential in cancer treatment.

Type	Natural Compounds	Anticancer chemotherapeutics	*in vitro*	Experimental models *in vivo*	Dose	Mechanism	Ref
Polyphenol	Curcumin	5-fluorouracil	Organism: human Cell lines: HCT116 and HCT116R Tissue: colon Origin: tumoral	-	Co-treatment regimen: Curcumin 5 μM and 5-Fluorouracil 0.8nM, 0.1nM in HCT116 and HCT116R cells respectively	Down-regulation of NF-κB activation and NF-κB-regulated gene products	[6]
Polyphenol	Curcumin	5-fluorouracil	Organism: human Cell lines: SW480, HCT116 Tissue: colon Origin: tumoral	Organism: mice Strain: athymic nude mice Cell line used for the xenograft model: HCT116-5-FU	Natural product: Curcumin 10 μM Chemotherapeutic agent: 5-Fluorouracil 10 µM Animal experiments: 5-Fluorouracil (20 mg/kg once every 2 days) and Curcumin (50 mg/kg daily) or (iv) 5-Fluorouracil and Curcumin.	Up-regulation of EMT-suppressive miRNAs.	[12]
Polyphenol	Curcumin	5-fluorouracil	Organism: human Cell lines: MKN45, AGS, GES-1Tissue: gastric Origin: tumoral and non-tumoral	Organism: mice Strain: nude mice Cell line used for the xenograft model: MKN45	Natural product: Curcumin Chemotherapeutic agent: 5-Fluorouracil Co-treatment regimen: 5-Fluorouracil and Curcumin (2:1, mol/mol)	Down-regulation of COX-2 and NF-κB pathway.	[13]
Polyphenol	Curcumin	5-fluorouracil and oxaliplatin (FOLFOX)	Organism: human Cell lines: CRLM, CSC Tissue: colon Origin: tumoral	-	Natural product: Curcumin Chemotherapeutic agent: FOLFOX Co-treatment regimen: Curcumin 5μM, Oxaliplatin 2 μM + 5-Fluorouracil 5 μM and Curcumin 5 μM + Oxaliplatin 2 μM + 5-Fluorouracil 5 μM	Downregulated expression of pluripotent stem cell markers Oct3-4, AFP and HNF/FoxA2 at 24 hours, and Nanog, Otx2 and VEGFR2 at 72 hours.	[16]
Polyphenol	Curcumin	Cisplatin	Organism: human Cell lines: 253J-Bv, T24Tissue: bladder Origin: tumoral	Organism: mice Strain: nude mice Cell line used for the xenograft model: 253J-Bv	Natural product: Curcumin Chemotherapeutic agent: Cisplatin Co-treatment regimen: Curcumin 10 μM and Cisplatin 10 μM	Activation of ERK1/2 mediated by ROS	[53]
Polyphenol	Curcumin	Imatinib	-	A case report patient with metastatic chemoresistant Adenoid cystic carcinoma	Imatinib 400 mg/day and intravenous curcumin 225 mg/m2 twice a week	Inhibition of NF-κB and mTOR pathways	[92]
Polyphenol	Curcumin	Doxorubicin	Organism: human Cell lines: MCF‑7/DOX,MDA‑MB‑231/DOX Tissue: breast Origin: tumoral	-	Natural product: Curcumin Chemotherapeutic agent: Doxorubicin Co-treatment regimen:10 µM Curcumin with 30 µM Doxorubicin	Inhibition of ATPase activity of ABCB4 without altering its protein expression	[108]
Polyphenol	Resveratrol	5-fluorouracil	Organism: human Cell lines: HCT116 and HCT116R Tissue: colon Origin: tumoral	-	Natural product: Resveratrol 5 μM Chemotherapeutic agent: 5-Fluorouracil Co-treatment regimen: Resveratrol 5µM with 5-Fluorouracil 1nM and TNF-β (10 ng/mL) or TNF-α (10 ng/mL)	Modulation of TNF-β signaling pathway, induction of apoptosis, suppression of NF-κB activation	[7]
Polyphenol	Resveratrol	5-fluorouracil	Organism: human Cell lines: B16 Tissue: skin Origin: tumoral	Organism: mouse Strain: Balb/c nu/nu mice Cell line used for the xenograft model: B16	Natural product: Resveratrol 5 μM Chemotherapeutic agent: 5-Fluorouracil Co-treatment regimen: 25 μM Resveratrol and 20 μM 5-Fluorouracil in combination	Inhibition of cell proliferation and reduction of tumor growth associated with changes in the expression levels of AMPK, VASP and VEGF	[26]
Polyphenol	Resveratrol	Cisplatin	Organism: human Cell lines: A549 Tissue: basal alveolar epithelial Origin: tumoral	-	Natural product: Resveratrol 2.5 µM Chemotherapeutic agent: Cisplatin 20 µM Co-treatment regimen: Resveratrol 2.5 µM and Cisplatin 20 µM for 24 h	Induction of apoptosis via modulating autophagic cell death	[39]
Polyphenol	Resveratrol	Cisplatin	Organism: human Cell lines: C3A, SMCC7721 Tissue: liver Origin: tumoral	-	Natural product: Resveratrol 12.5 μg/ml Chemotherapeutic agent: Cisplatin 20 µM Co-treatment regimen: Resveratrol 12.5 μg/ml and Cisplatin 0.625 μg/ml for 24h	Apoptosis-dependent mechanism and glutamine metabolism inhibition	[54]
Polyphenol	Resveratrol and didox (DID)	Doxorubicin	Organism: human Cell lines: HCT116 Tissue: colon Origin: tumoral	-	Natural product: Resveratrol Chemotherapeutic agent: Doxorubicin Co-treatment regimen: Combination of Doxorubicin with Resveratrol and DID in HT-29 were 0.47 ± 0.02 μM and 0.29 ± 0.04 μM, respectively	Increased *BAX* and *TP53* gene expression	[70]
Polyphenol	(E)-3-(3,5-dimethoxyphenyl)-1-(2-methoxyphenyl)prop-2-en-1-one (DPP-23)	Cisplatin	Organism: human Cell lines: HN3, HN4, and HN9 Tissue: oral keratinocytes or fibroblasts Origin: non-tumoral, tumoral	-	Natural product: DPP-23 Chemotherapeutic agent: Cisplatin Co-treatment regimen: DPP-23 in doses of 2.5 to 10 μmol/L and Cisplatin 10 μmol/L	ROS-mediated apoptotic cell death	[40]
Polyphenol	Caffeic acid	Cisplatin	Organism: human Cell lines: A2780, A2780cisR Tissue: ovarian Origin: tumoral	-	Natural product: Caffeic Acid 10 µM Chemotherapeutic agent: Cisplatin 20 µM Co-treatment regimen: 5:50 µM Cisplatin/Caffeic acid	Apoptosis induction via caspase-3 activation	[41]
Polyphenol	Luteolin	Cisplatin	Organism: human Cell lines: CAOV3/DDP Tissue: ovarian Origin: tumoral	Organism: mice Strain: BALB/c nude mice Cell line used for the xenograft model: Cisplatin resistant cell line CAOV3/DDP	Natural product: Luteolin 10, 50, 100 μM Chemotherapeutic agent: Cisplatin 2 μg/ml Co-treatment regimen: Luteolin 0, 10, 50, 100 μM and Cisplatin in combination for 48 h	Downregulation of Bcl-2 expression, reduction of tumor growth and apoptosis induction	[55]
Polyphenol	Epigallocatechin-3-gallate	Cisplatin	Organism: human Cell lines: A549, A549R, H460 and H1299 Tissue: basal alveolar epithelial cells Origin: tumoral	Organism: mouse Strain: nude mouse Cell line used for the xenograft model: A549	Natural product: Epigallocatechin-3-gallate 20μM Chemotherapeutic agent: Cisplatin 10μM In vivo study: control (normal saline, 0.1 ml/10g), Epigallocatechin-3-gallate 20mg/kg, Cisplatin 5mg/kg, and Epigallocatechin-3-gallate 20 mg/kg)with Cisplatin 5mg/kg	Upregulated *CTR1* expression and increased intracellular uptake of Cisplatin	[65]
Polyphenol	(-)-Epigallocatechin-3-gallate	Cisplatin	Organism: human Cell lines: H1299 and H460 Tissue: lung Origin: tumoral	Organism: mice Strain: female athymic nude mice Cell line used for the xenograft model: H460	Natural product: 15μM. In vivo stusy Pro-Epigallocatechin-3-gallate at 60 mg/kg. Chemotherapeutic agent: Cisplatin Co-treatment regimen: Cisplatin 4mg/kg pharmaceutical grade, three times weekly by IP injection	Inhibition of DNA repair mechanism by downregulation of ERCC1/XPF activity	[69]
Polyphenol	Epigallocatechin-3-gallate	Doxorubicin	Organism: human Cell lines: U2OS and SaoS 2 Tissue: bone Origin: tumoral	-	Co-treatment regimen: For U2OS and SaoS2 cells Doxorubicin 1 and 2.5 μM, Doxorubicin with Epigallocatechin-3-gallate (0 μM + 20μg/ml, 1μM+ 20 μg/ml and 2.5μM+ 20 mg/ml)	Reduction of autophagy by downregulation of SOX2OT variant 7 gene expression	[77]
Polyphenol	Eupatorin and Salvigenin	Doxorubicin	Organism: human Cell lines: HT-29, SW948 and HFFF-2 Tissue: colon Origin: tumoral	-	Co-treatment regimen: Salvigenin (25- 200 µM), Eupatorin (25- 200 µM) and Doxorubicin (1- 20 µM)	Apoptosis induction by increased Bax/Bcl-2 ratio, caspase-3 expression and PARP cleavage	[78]
Polyphenol	Urolithin A	Oxaliplatin	Organism: human Cell lines: HCT116 (WT, p53−/− and p21−/−)Tissue: colon Origin: tumoral	-	Natural product: Urolitin A 19 μM Chemotherapeutic agent: Oxaliplatin 1.6 μM	p53 stabilization and p53 target gene expression which results in cell cycle regulation and glycolysis inhibition	[99]
Alkaloid	Neferine and isoliensinine	Cisplatin	Organism: human Cell lines: HCT-15 Tissue: colon Origin: tumoral	-	Natural product: Neferine 6 μM, Isoliensinine 8 μM Chemotherapeutic agent: Cisplatin 15 μM Co-treatment regimen: different concentrations of Neferine 6 μM + 15 μM of Cisplatin, 8 μM of Isoliensinine + 15 μM of Cisplatin	Increased intracellular uptake of Cisplatin and mitochondrial apoptosis induction by ROS-mediated mechanism	[37]
Alkaloid	Neferine	Cisplatin	Organism: human Cell lines: A549 Tissue: lung Origin: tumoral	-	Natural product: Neferine 0-20 μM, Chemotherapeutic agent: Cisplatin 10-20 μM Co-treatment regimen: Neferine 10 μM and Cisplatin 10-20 μM	mTOR inhibition and ROS-mediated autophagy induction	[38]
Alkoloid	Berberine	Cisplatin	Organism: human Cell lines: OVCAR3 Tissue: ovarian Origin: tumoral	-	Natural product: Berberine 50, 100, 200, 500 μM Chemotherapeutic agent: Cisplatin 5 mg/L Co-treatment regimen:: Berberine 100 μM and 5 mg/L of Cisplatin	Inhibition of proliferation and enhanced apoptotic and necroptotic cell death	[47]
Alkaloid	Berberine	Cisplatin	Organism: human Cell lines: MCF-7 Tissue: breast Origin: tumoral	-	Natural product: Berberine Chemotherapeutic agent: Cisplatin Co-treatment regimen:Berberine 13 μM and Cisplatin 3.3 μM	Regulation of DNA repair machinery promoting DNA breaks and apoptotic cell death	[48]
Alkaloid	Emetine	Cisplatin	Organism: human Cell lines: A2780 and A2780 Cisplatin resistant Tissue: ovarian Origin: tumoral	-	Natural product: Emetine 0.01–0.21 μM Chemotherapeutic agent: Cisplatin 0.18–46.16 μM Co-treatment regimen: Emetine 0.01–0.21 μM/Cisplatin 0.18–46.16 μM	Reduced cell viability	[49]
Alkaloid	Tetrandrine	Cisplatin	Organism: human Cell lines: MDA-MB-231 Tissue: breast Origin: tumoral	-	Natural product: Tetrandrine 8–128 μM Chemotherapeutic agent: Cisplatin 10–166 μM Co-treatment regimen: Tetrandrine 8-52 μM and Cisplatin 4.5-17.8 μM	Apoptosis induction by ROS-mediated mechanism	[50]
Alkaloid	Dendrobine	Cisplatin	Organism: human Cell lines: A549 Tissue: lung Origin: tumoral	Organism: mice Strain: BALB/c Cell line used for the xenograft model: A549	Natural product: Dendrobine 0-15 ug/ml Chemotherapeutic agent: Cisplatin 1 μg/ml Co-treatment regimen: Dendrobine 1-10 ug/ml and 1 ug/ml of Cisplatin Animal experiment: Cisplatin 2.5 mg/kg/week and Dendrobine 50 mg/kg/d were	Induction of JNK/p38 stress signaling pathways and pro-apoptotic Bax and Bim protein activation	[51]
Alkaloid	Sophoridine	Cisplatin	Organism: human Cell lines: NCI-H460, NCI-H1299, and A549 Tissue: lung Origin: tumoral	Organism: mice Strain: BALB/c Cell line used for the xenograft model: NCI-H460	Natural product: Sophoridine 20 μg/mL Chemotherapeutic agent: Cisplatin 6 μM Co-treatment regimen: Sophoridine 20 μg/mL combined with Cisplatin 6 μM Animal experiment: in Sophoridine 16.9 mg/kg and cisplatin 4.8 mg/kg	Enhancement of Cisplatin sensitivity through the activation of p53 and Hippo signaling pathways	[52]
Alkaloid	Piperlongumine	Doxorubicin	Organism: human Cell lines: DU-145 Tissue: prostate Origin: tumoral	-	Natural product: Piperlongumine 0.1, 0.5, 1 μM Chemotherapeutic agent: Doxorubicin 0.01, 0.05, 0.1 μM Co-treatment regimen: Piperlongumine 0.1-1 μM and Doxorubicin 0.01-0.1 μM	Antiproliferative and pro-apoptotic effect with the up-regulation of cleaved PARP and caspase-3 proteins	[72]
Alkaloid	Coralyne	Paclitaxel	Organism: human Cell lines: MCF-7 and MDA-MB-231Tissue: breast Origin: tumoral	-	Natural product: Coralyne 6.25–100 μM Chemotherapeutic agent: Paclitaxel 5–40 nM Co-treatment regimen: Coralyne 5-20 μM and Paclitaxel 0.005-0.020 μM	Inhibition of ki-67 proliferation marker expression and up-regulation of the pro-apoptotic protein Bax	[86]
Alkaloid	Nuciferine	Paclitaxel	Organism: human Cell lines: HCT-8, HCT-8/T, A549(NSCLC) and A549/T Tissue: colorectal and lung Origin: tumoral	Organism: mice Strain: BALB/c Cell line used for the xenograft model: A549/T	Natural product: Nuciferine 48, 24, and 4 μM Chemotherapeutic agent: Paclitaxel Co-treatment regimen: Nuciferine 4-48 μM and Paclitaxel 0.01-100 μM Animal treatments: Paclitaxel 10 mg/kg and Nuciferine 7.5 mg/kg NF	Inhibition of PI3K/AKT/ERK pathways, which results in supressed Nrf2, HIF-1α, P-gp and BCRP expression	[87]
Alkaloid	Piperine	Paclitaxel	Organism: human Cell lines: SKOV-3Tissue: ovarian Origin: tumoral	-	Natural product: Piperine 10, 20, 30, 40 µM Chemotherapeutic agent: Paclitaxel 1, 5, 10, 15, 20 nM Co-treatment regimen: Piperine 10 µM and Paclitaxel 5 nM	ROS-medianted apoptosis induction	[88]
Alkaloid	Piperlongumine	Paclitaxel	Organism: human Cell lines: INT-407 and HCT-116 Tissue: intestinal Origin: tumoral	-	Natural product: Piperlongumine 1, 2.5 and 5 μM Chemotherapeutic agent: Paclitaxel 0.1, 0.5 and 1 μM Co-treatment regimen: Piperlongumine 1-5 μM and Paclitaxel 0.1 to 1 μM	ROS-medianted cell death	[89]
Alkaloid	Piperlongumine	Docetaxel	Organism: human Cell lines: MDA-MB-231, HCC 70, HCC 1806, HS578T, MDA-MB-468, and caco-2 Tissue: breast and colon Origin: tumoral	Organism: rat Strain: Sprague–Dawley for *in vivo* pharmacokinetic analysis	Natural product: Piperlongumine 0.2-20 μM Chemotherapeutic agent: Docetaxel 0.01-2 μM Co-treatment regimen for pharmacokinetic analysis: Docetaxel 10mg/kg and Piperlongumine 50mg/kg and PPL 10 and 50 mg/kg, respectively	Reduction of Docetaxel efflux i*n vitro* and enhancement of Docetaxel bioavailability *in vivo,* modulation of proliferation markers expression	[94]
Terpenoid	Oridonin	Cisplatin	Organism: humanCell lines: A549 and B2b Tissue: human bronchial epithelium cell Origin: tumoral	Organism: mice Strain: C57BL/6 WT for nefrotoxicity analysis	Natural product: Ori 5, 10, and 20 μM Chemotherapeutic agent: Cisplatin 10, 20 μM Co-treatment regimen: Ori 10, 20 μM and CDDP 20 μM Co-treatment regimen for nefrotoxicity analysis: Cisplatin 20mg/kg intraperitoneal injection for 3 days to induce acute kidney injury and Oridonin 20 mg/kg injection simultaneously	Induction of apoptosisthrough AMPK/Akt/mTOR-dependent autophagosome activation	[36]
Terpenoid	Cucurbitacin B	Cisplatin	Organism: human Cell lines: A2780, A2780CP Tissue: ovarian cell Origin: tumoral	-	Natural product: Cucurbitacin B 0.5, 1, 2, 4, and 6 μM Chemotherapeutic agent: Cisplatin 20 μM Co-treatment: Cucurbitacin B 2 μM for 24 h then further incubated for another 24 h with 20 μM cisplatin.	Decrease of cell viability through pERK1/2 and pSTAT3 levels modulation	[42]
Terpenoid	Cucurbitacin B	Cisplatin	Organism: mouse Cell lines: MB49 Tissue: bladder cell Origin: tumoral	Organism: mouse Strain: C57BL/6 Cell line used for the xenograft model: MB49	Natural product: Cucurbitacin B 0.01–50 μM Chemotherapeutic agent: Cisplatin 0.5–50 μM Co-treatment regimen: Animal experiments: Cucurbitacin B 0.5mg/kg was injected intraperitoneally three times a week and Cisplatin 2 mg/kg was injected intraperitoneally twice a week	Reduction of tumor growth through caspase-dependent/independent apoptotic and autophagic pathways	[43]
Terpenoid	Borneol	Doxorubicin	Organism: human Cell lines: U251, U87 Tissue: gliomacell Origin: tumoral	Organism: mice Strain: male nude mice Cell line used for the xenograft model: U251	Natural product: Borneol 0.1–1 μM Chemotherapeutic agent: Doxorubicin 20–160 μM Co-treatment regimen: Borneol 80 μM with or without 0.4 μM/0.8 μM Doxorubicin	Borneol enhances the intracellular uptake of Doxorubicin and activates ROS production	[71]
Terpenoid	Borneol	Temozolomide	Organism: human Cell lines: U251 Tissue: gliomacell Origin: tumoral	Organism: mice Strain: Balb/c Cell line used for the xenograft model: U251	Natural product: Borneol 5–80 µg/mL Chemotherapeutic agent: Temozolomide 10–160 µM Co-treatment regimen: Temozolomide 20 and 40 µM and Borneol 40 and 80 µg/m)	ROS-medianted cell death	[96]
Terpenoid	Vielanin k	Doxorubicin	Organism: human Cell lines: MCF-7, MCF-7/MDR and MCF-10A Tissue: breast and mammarycell Origin: tumoral and non-tumoral	-	Co-treatment regimen: Vielanin k 5, 10, or 15 or 10 μM with 0.5 μM (MCF-7) or 5 μM (MCF-7/MDR) Doxorubiccu for 4 h.	Ativation of endoplasmic reticulum estress and mitochondrial apoptosis via IRE1α-TRAF2-JNK signaling	[73]
Terpenoid	Vielanin P	Doxorubicin	Organism: human Cell lines: MCF-7/MCF-7/MDR; The K562 and K562/ADR Tissue: breast and myelegenous leukemiacell Origin: tumoral	-	Natural product: Vielanin P Chemotherapeutic agent: Doxorubicin Co-treatment regimen: 5, 10, or 15 μM Vielanin P with 0.5 μM (MCF-7) or 5 μM (MCF-7/MDR) Doxorubicin for 4 h.	Induction of Doxorubicin accumulation through the reduction of *MRP1* expression via PI3K/Nrf2 signaling	[74]
Terpenoid	β-caryophyllene oxide and trans-nerolidol	Doxorubicin	Organism: human Cell lines: MDA-MB-231 and MCF7 Tissue: breast cell Origin: tumoral	Organism: mice Strain: NMRI Cell line used on the xenograft model: Ehrlich tumors (EST)	Natural product: β-caryophyllene oxide 5-500 μM and trans-nerolidol 5-500 μM Chemotherapeutic agent: Doxorubicin 0.1-3 μM Co-treatment regimen: Doxorubicin 3 μM + trans-nerolidol 225 μM and Doxorubicin 3 μM + β-caryophyllene oxide 225 μM	Antiproliferative effect potentiation and increased Doxorubicin intracellular accumulation	[79]
Terpenoid	Ginkgolide B	Gemcitabine	Organism: human Cell lines: BxPC-3, CAPAN1, PANC1 and MIA PaCa-2 Tissue: pancreatic cell Origin: tumoral	Organism: mice Strain: nude mice Cell line used for the xenograft model: CAPAN1	Natural product: Ginkgolide B 0–500 μM Chemotherapeutic agent: Gemcitabine 10 or 20 nM Co-treatment regimen: Gemcitabine 0–200 nM and Ginkgolide B 25, 100 or 400 μM for six days	Supression of NF-кB activity and potentiation of antiproliferative effects	[98]
Terpenoid	Pachymic acid and dehydrotumulosic acid	Doxorubicin and Cisplatin	Organism: human Cell lines: HepG-2; MCF and MCF/ADR DOX; A549 and A549/CDDP Tissue: liver, breast and lung cell Origin: tumoral	Organism: mice Strain: BALB/c Cell line used for the xenograft model: MCF/ADR	Natural product: Pachymic acid and Dihydrotumulosic acid Chemotherapeutic agent: Doxorubicin and Cisplatin Co-treatment regimen: Doxorubicin 2.5 mg/kg and Pachymic acid	Inhibition of the P-gp function and enhanced acumulation of Doxorubicin and potentiation of Doxorubicin biological effects	[109]

The inflammatory process has an essential role in the tumor microenvironment, which is key to the promotion of cancerous cells and thus to tumor progression [9]. Various studies have reported that proinflammatory cytokines act mainly due to the activation of NF-κB (*Nuclear Factor NF-Kappa-B*), a transcription factor that can induce the expression of various genes involved in the modulation of apoptosis and promotion of cellular transformation, progression, invasion, metastasis, survival, chemoresistance, radioresistance, and inflammation of early and late stage aggressive tumors. For this reason, different natural compounds with immunomodulatory activity have been reported as possible adjuvants to chemotherapy [10]. However, products of natural origin are not exempt from risk, so the adverse effect of phytochemicals, including their interactions with each other and with chemotherapy drugs, must be carefully considered.

**Figure 1:**
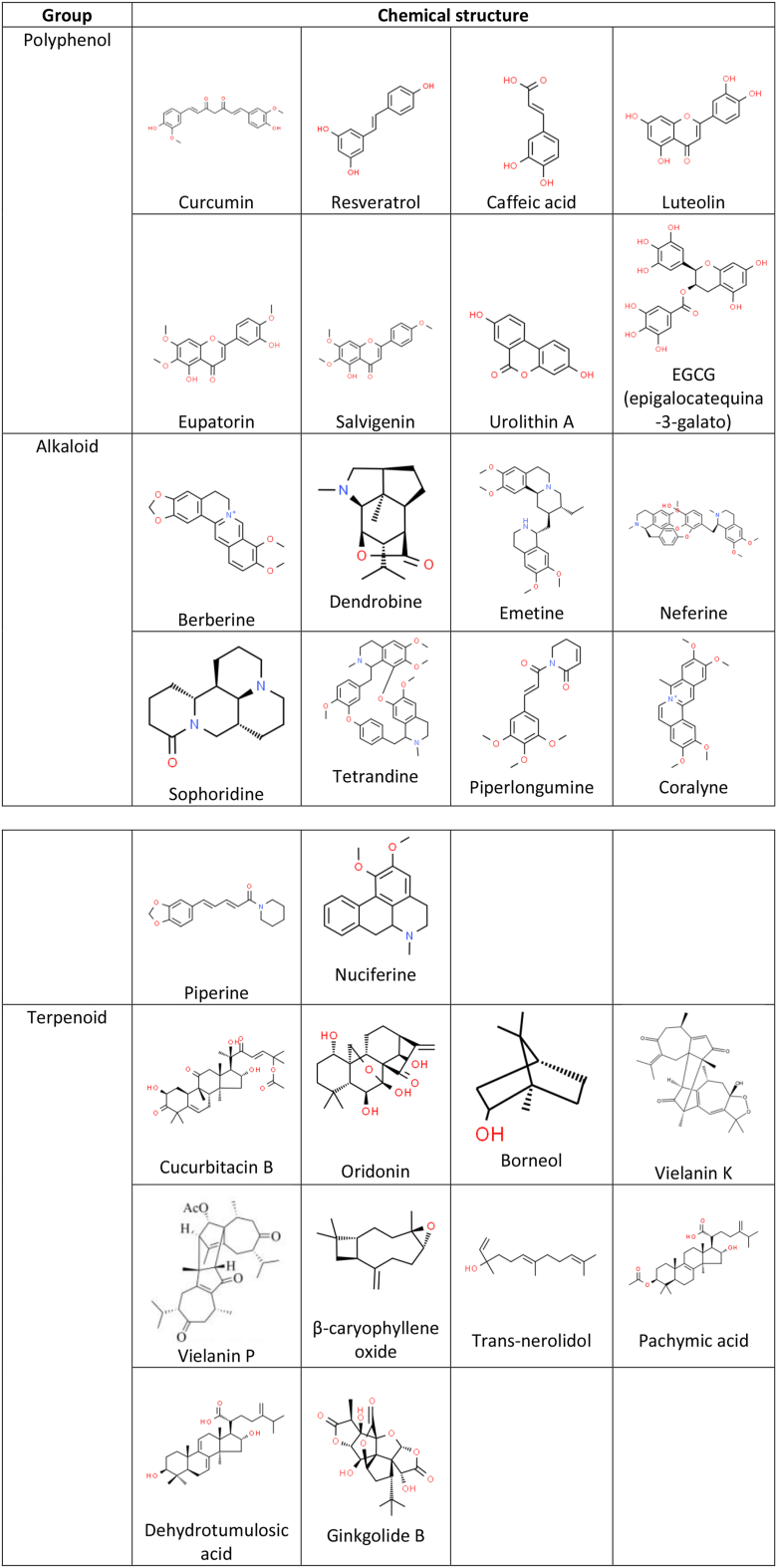
Chemical structures of natural compounds and chemotherapeutic agents reported in the review. Chemical structures were obtained from ChemSpider (https://www.chemspider.com) and PubChem (https://pubchem.ncbi.nlm.nih.gov).

Chemotherapy is the standard treatment for cancer. However, resistance of cancerous cells to a wide range of chemotherapeutic and targeted drugs has become a frequent occurrence; indeed, a high percentage of deaths among cancer patients are attributed directly or indirectly to drug resistance [11]. Thus, the development of strategies that overcomes cancer treatment resistance has become a considerable challenge; fortunately, some natural products with a wide range of chemical structures and pharmacological effects, have been reported to be effective against drug resistance in cancer therapy. Here, we review the influences of natural compounds such as polyphenols, alkaloids and terpenoids (Figure 1) on the potentiation of the biological effects and the reversal to resistance of chemotherapy drugs, e.g. 5-Fluorouracil (5-FU), Cisplatin (CDDP), Doxorubicin (DOX), Paclitaxel (PTX), among others, on *in vitro* and *in vivo* models of cancer.

## 2 Literature search

**Figure 2:**
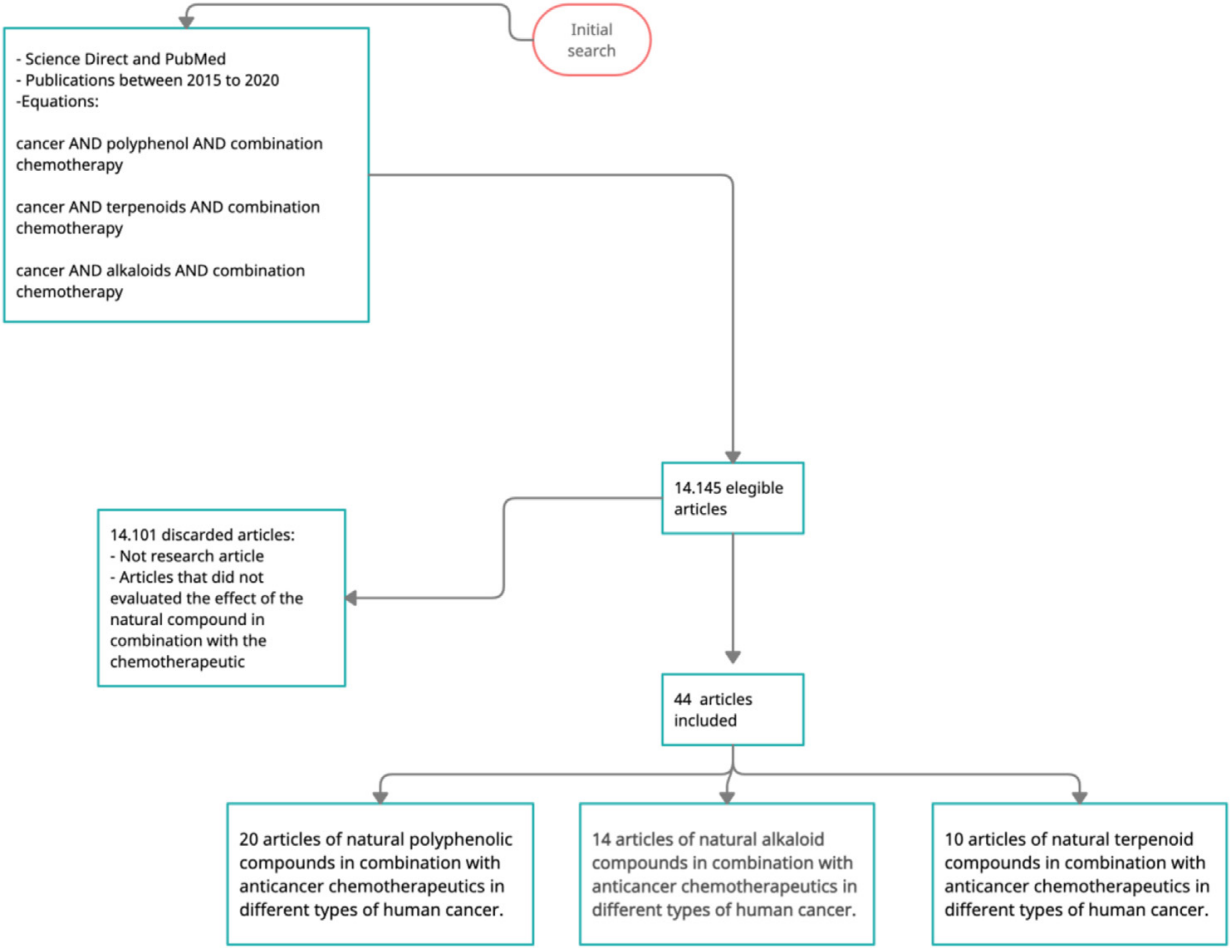
Literature search. Flow diagram used in the search equation to select the articles for the literature review.

This literature review was conducted with papers published between 2015 and 2020, using multiple search equation to find studies that includes these terms in the title and the abstract (Figure 2). This resulted in a preliminary list of 14.145 papers, but not original papers as well as those that not evaluated the effect of natural compounds in combination with chemoterapeutic drugs were discarded; likewise, studies without full text availability or writed in language different than english, were also discarded. In this manner, 44 papers met all the criteria and were used for the review (Figure 2).

### 2.1 5-Fluorouracil

5-FU is an antimetabolite used as a first-line chemotherapeutic agent for various types of cancer. However, its clinical application is limited by serious adverse effects such as gastrointestinal toxicity and bone marrow suppression. Moreover, many patients are refractory to 5-FU based treatment, reducing its efficacy [12, 13]. Curcumin (diferuloylmethane) is a polyphenol present in the rhizome of the plant *Curcuma longa* (also known as haldi or haridra in parts of Asia and as curry powder in the West). It has been consumed for centuries as a dietary component and is also used to combat various inflammatory diseases. In recent decades, a range of biological activities of curcumin have been widely reported, including antioxidant, anti-inflammatory, antimicrobial, antiviral and anticancer effects, the last of which has been widely described [14].

The most recent evidence suggests that curcumin acts as a chemosensitizer and radiosensitizer in various types of cancer [15]. It has been demonstrated that the combination of curcumin and 5-FU sensitizes colon cancer cells that are resistant to treatment, which results in a lower drug requirement for therapy [12]. Moreover, in studies where the chemotherapeutic oxaliplatin was included in combination with 5-FU and curcumin, significant inhibition in the proliferation of gastric cancer cells [13] and an increase in the apoptosis index [16] were observed. Similarly, Shakibaei et al. analyzed the effect of the combination of curcumin and 5-FU on HCT116 colon cancer cells and their isogenic clones that were resistant to treatment with 5-FU (HCT116R), finding an increase in apoptosis and a reduction in proliferation and formation of colonies [6]. Similar findings were reported using *in vitro* models of gastric cancer, where the combination of curcumin with 5-FU resulted in an increase in cellular toxicity and a reduction in the expression of COX-2 (*Cyclooxygenase-2*) and NF-κB (*Nuclear Factor NF-Kappa-B*); likewise, a reduction in tumor size were observed in nude-mice inoculated with MKN45 cells and exposed to 5-FU and curcumin, when compared with mice only treated with 5-FU [13].

Resveratrol (3,5,41- trihidroxiestilben) is a natural ocurring non-flavonoid polyphenol present in various plants and fruits from the *Vitis* and *Vaccinium* genders, such as peanuts and grapes. Its main action is to provide protection from ultraviolet radiation, oxidative stress and fungal infections. Studies have shown that resveratrol has anti-inflammatory and antitumoral effects in various types of cancer [17, 18, 19, 20]. Moreover, it has been demonstrated that resveratrol has a chemosensitization effect on cancer cells when applied in combination with drugs used for chemotherapy, inducing apoptosis and suppressing signalling pathways that promote inflammation. Buhrmann et al. evaluated the modulatory effect of resveratrol, both as a monotherapy and in combination with 5-FU in 3D colon cancer cell models (HCT116) and their isogenic clones resistant to 5-FU (HCT116R), in the presence of an inflammatory microenvironment mediated by the addition of the proinflammatory cytokines TNF-β (*Tumor Necrosis Factor Beta*) and TNF-α (*Tumor Necrosis Factor Alfa)*. In this manner they found that the combination of resveratrol with 5-FU generated greater inhibition of the invasion capacity of HCT116 and HCT116R, even in the presence of TNF-β; moreover, this combination produced the modulation of the proinflammatory NF-κB pathway, causing sensitization of the colon cancer cells to treatment with 5-FU [7].

Additionally, resveratrol has been shown to have the potential to inhibit angiogenesis in different cancer models [21, 22, 23], as well as the expression of HIF-1α (*Hypoxia-Inducible Factor 1 alpha*) and VEGF (*Vascular Endothelial Growth Factor*), through various mechanisms such as the inhibition of PKB (*Protein Kinase B*), the activation of MAPKs (*Mitogen-Activated Protein Kinases*) and the suppression of capillary formation [24, 25]. Lee et al. analyzed the antiangiogenic effects of resveratrol and 5-FU, both as a monotherapy and in combination, on a murine melanoma model. They found that the combined treatments reduced the number of microvascular vessels in comparison with the control group, suggesting that this would effectively reduce angiogenesis. Moreover, they demonstrated that the combination of resveratrol and 5-FU reduced the proliferation capacity and size of the tumors, which could be explained by changes in the expression levels of AMPK (*AMP-activated Protein Kinase*), VASP (*Vasodilator Stimulated Phosphoprotein*) and VEGF [26].

### 2.2 Cisplatin

CDDP is an inorganic platinum agent currently used in standard chemotherapy to treat different types of cancer. The main chemotherapeutic mechanisms of CDDP are based on induction of oxidative stress, DNA damage and apoptosis; however, resistance to CDDP treatment has been observed, which limits its use in clinical practice. Various studies have shown that PI3K (*Phosphatidylinositol 3-Kinase*), the MAPKs pathways and proteins involved in the modulation of apoptosis such as Bax (*BCL2 Associated X Protein*) and Bcl-2 (*BCL2 Apoptosis Regulator*), plays significant roles in the resistance to CDDP [27, 28, 29, 30]. However, it has also been demonstrated that natural compounds such as resveratrol, neferine and oridonin can inhibit cellular proliferation and induce apoptosis through modulation of the PI3K/Akt/mTOR (*Phosphatidylinositol 3-Kinase/AKT Serine/Threonine Kinase/Mechanistic Target Of Rapamycin Kinase)* and MAPKs pathways [31, 32, 33, 34, 35]. Interestingly, oridonin has been also reported to have a protective effect on the nephrotoxicity induced by CDDP [36].

Recent studies on *in vitro* models of colon and lung cancer have found that the combination of neferine and CDDP promotes intracellular accumulation of the chemotherapeutic. This results in an increase in apoptosis and autophagy induced by ROS (*Reactive Oxygen Species*), through modulation of the MAPKs and PI3K/Akt/mTOR pathways, and activation of the independent, non-canonical pathway of Beclin-1 and PI3K CIII [37, 38]. Similarly, in A549 cells resveratrol in combination with CDDP induces autophagia through negative regulation of the PI3K/Akt/mTOR pathway, as well as apoptosis through modulation of the expression of Bax and Bcl2 [39].

Kim et al. evaluated the effect of CDDP in combination with a mixture of DP-23 (E)-3-(3.5-d) - dimethoxyphenyl 1- (2- methoxyphenyl) prop-2-in-1) polyphenols on cisplatin-sensitive and cisplatin-resistant head and neck cancer cell lines. Specifically, they shown the increase in the production of ROS and the inhibition of Nrf2 (*Nuclear Factor Erythroid 2-Related Factor 2*), which results in cell death of cancer cells including cisplatin-resistant cells lines, but not of normal cells, suggesting a selective effect. The importance of the modulation of Nrf2 is based on the fact that the signalling pathway of Nrf2 induces the expression of GST (*Glutathione S-Transferase*) and GSR (*Glutathione Reductase*), enzymes that have been involved in resistance to CDDP [40, 41]. Sirota et al. evaluated the effect of caffeic acid in combination with CDDP on *in vitro* models of ovarian cancer, on the basis that caffeic acid is an abundant polyphenol in many foods and, moreover, because it has been shown to inhibit the activity of GST and GSR. In this study, it was found that combined treatments potentiates the cytotoxicity induced by CDDP, as well as the quantity of platinum bonded to nuclear DNA, suggesting that caffeic acid has a sensitizing effect [41].

Cucurbitacin B, a tetracyclic triterpenoid derived from plants from the Cucurbitaceae family, is highly-studied due to its antitumoral effect. This terpenoid, in combination with CDDP, leads to increased cytotoxicity in both CDDP-sensitive and CDDP-resistant bladder cancer cells. This effect were mediated by an increase in ROS and the modulation of the STAT-3 (*Signal Transducer And Activator Of Transcription*) and ERK1/2 (*Extracellular Signal-Regulated Kinase1/2*) signalling pathways, and reducing the levels of Dyrk1B (*Dual Specificity Tyrosine Phosphorylation Regulated Kinase 1B*) [42]. Similarly, this combined treatment significantly reduced tumoral growth through activation of caspase-dependent and caspase-independent autophagic and apoptotic pathways in bladder cancer cells [43].

Berberine (BBR) is an isoquinoline alkaloid extracted from a diverse range of medicinal plants and used in the treatment of various diseases [44]. Recent studies indicate that BBR has activity against cancers of the liver, breast, ovaries, glioblastoma, and other types of cancer, based on a variety of biological mechanisms [45, 46]. Comparing the use of BBR and CDDP separately and in combination, it has been found that the combination of these compounds has a greater effect on the inhibition of cell growth in ovarian and breast cancer models, through the regulation of cell proliferation and apoptosis/necrosis induction. This was demonstrated by a greater expression and activation of caspase 3 and 8, RIPK3 (*Receptor Interacting Serine/Threonine Kinase 3*) and MLKL (*Mixed Lineage Kinase Domain Like Pseudokinase*), in OVCAR3 and three primary ovarian cancer cell lines derived from patients [47]. Similarly, it has been observed that low doses of BBR can sensitize breast cancer cells lines to CDDP, positively regulating caspases 3 and 9, negatively regulating the expression of the Bcl-2 protein and, most importantly, increasing the DNA damage caused by CDDP while reducing the level of cellular PCNA (*Proliferating Cell Nuclear Antigen*) [48]. Also, synergistic activity has been found between CDDP and other alkaloids such as emetine and tetrandrine in cell lines of ovarian and breast cancer, leading to an increase in ROS production and activation of caspase-3, 7 and 8 [49,50]. Moreover, chemosensitization to CDDP has been shown to be enhanced by alkaloids such as dendrobine and sophoridine in lung cancer cells lines. Similarly, dendrobine has been found to mitigate the reduction in bodyweight and cardiotoxicity induced by CDDP in animals models [51, 52].

Bong Hee et al. evaluated the effect of combining CDDP and curcumin in 253J-Bv and T24 cell lines and animal bladder cancer models. The results of this study showed increased inhibition of the migration of cancer cells, increased levels of phosphorylated MEK (Mitogen-activated Protein Kinase) and regulation of the protein levels of phosphorylated ERK1/2. Moreover, an increase in the level of apoptosis mediated by the generation of ROS and the phosphorylation of ERK were observed, in comparison with the apoptosis percentage of the control group or the individual treatments. Additionally, in the animal model a significant reduction was found in tumor volume with the combined treatment and, moreover, no difference was found in the bodyweight of animals, indicating a good tolerance to treatment [53].

In another study, it was evaluated the sensitization caused by combined treatment with resveratrol and CDDP in C3A, SMCC7721 and LO2 human hepatoma cells (normal hepatic cells). In C3A and SMCC7721 cells, this combined treatment increased apoptosis and had a greater cytotoxic effect than the compounds used individually, mainly due to the increase in ROS production. It is important to highlight that, in this study, resveratrol did not increase the inhibitory effect of CDDP on LO2 growth, which indicates that the combination of resveratrol with CDDP is selective, and does not affect cells of non-tumoral origin [54]. Another polyphenol that has been used in combination with CDDP is luteolin. This has been evaluated in an *in vitro* model of CDDP-resistant ovarian cancer cells (CAOV3/DDP) and it was found a dose-dependent inhibition of cellular proliferation, increased antiproliferative effect in the CDDP-resistant cells, and an increase in cellular apoptosis. Moreover, it was found that the combination of CDDP with luteolin increased the negative regulation of Bcl-2 expression, and increased the inhibition of the migration and invasion of CAOV3/DDP cells [55].

EGCG is the catechin with the highest concentration in green tea. It has been reported that EGCG can promote sensitivity to traditional cancer medicines [56, 57, 58] and reverse resistance to various drugs [59]. Jiang and et al. evaluated the effect of CDDP treatments in combination with EGCG on the expression of the *CTR1* solutes carrier (*Copper Transporter 1*) in ovarian cancer models, which is important, considering that CTR1 has been associated with the regulation of intracellular CDDP levels. Therefore, it has been proposed that the increase in *CTR1* expression could promote chemosensitivity to CDDP [60, 61, 62, 63, 64]. In this regard, the researchers found that EGCG induces *CTR1* expression at the mRNA and protein level and, moreover, inhibits its degradation. Additionally, they found that the combination of EGCG with CDDP increases the intracellular accumulation of the chemotherapeutic and promotes the sensitization of the SKOV3 and OVCAR3 cancer cells to treatment, and the reduction in tumor growth in animal models [8].

In another study carried out by the same researchers, the role of non-coding RNAs in the regulation of *CTR1* expression in the treatment of lung cancer cells with CDDP and EGCG was evaluated. The researchers found that ECGC induces the expression of *CTR1* and long non-coding RNA (lncRNA) NEAT1, and reduces the expression of microRNA mir-98-5p, which have been proposed as possible positive and negative regulators of *CTR1* expression, respectively. The results shown that the combined treatment inhibits the growth of tumors in animal lung cancer models, reduces the expression of Ki-67, and promotes the capture of CDDP in lung cancer cell lines, confirming that the positive regulation of *CTR1* expression is a mechanism that promotes chemosensitization to CDDP [65].

As mentioned above, the main mechanism of platinum-based drugs is the induction of DNA damage. It has been suggested that resistance to these chemotherapeutics can be generated by, among other mechanisms, increased ability of cancer cells to repair this damage, thereby considerably reducing the efficacy of the drug [66, 67, 68]. For this reason, studies have been carried out with the aim of identifying molecules that inhibit the activity of the repair mechanisms. One such study found that the small molecules NSC143099 and NSC16168 can inhibit the activity of the ERCC1-XPF heterodimer, an endonuclease that is essential in multiple DNA repair pathways in mammal cells, which results in the potentiation of cytotoxicity induced by CDDP in tumor cells and the inhibition of tumor growth in animal models [68]. Additionally, the same researchers analyzed the effect of the combination of EGCG and CDDP using *in vitro* lung cancer models, considering that the structure of EGCG has a 90% similarity to that of NSC143099. In this way, it was demonstrated that ECGC is a potent inhibitor of ERCC1-XPF activity, which leads to sensitization of tumor cells to CDDP, thus increasing cell death and reducing proliferation [69].

### 2.3 Doxorubicin

DOX is a chemotherapeutic widely used due to its efficacy in a large range of tumors, such as carcinomas, sarcomas and hematologic neoplasms; however, it has shown limited efficacy in colorectal cancer. This effect is mainly attributed to the overexpression of the P-glycoprotein (P-gp), which is an ADP-dependent pump that promotes the efflux of xenobiotics such as DOX from cells, causing chemotherapeutic treatment failure [70]. Pachymic and dehydrotumulosic acid (PT) are triterpenoids extracted from *Poria cocos,* a Chinese medicinal herb of fungal origin, whose effects on the potentiation of chemotherapy have been evaluated. Yanan Li et al. proposed a liposomal co-delivery system for the encapsulation of DOX and PT, in order to evaluate its effects on breast cancer models. They found that co-administration of PT enhance the sensitivity of drug-resistance MCF cells to DOX and improved the anti-tumor effect of DOX in mice subcutaneously injected with DOX-resistant breast cancer tumor cells, effects that could be explained by the modulation of the expression and function of P-gp [70].

Khalee et al. evaluated the sensitizer effect of two polyphenols, resveratrol and didox (DID), on treatment against colon cancer in combination with DOX. The researchers found that intracellular retention of DOX increases due to the inhibition of the efflux activity of P-gp, which is mediated by resveratrol and DID. In the same study, the effect of DOX treatment in combination with resveratrol or DID, on the gene expression of key apoptosis markers in HCT116 and HT29 cells was evaluated. In this regard, a significant increase in the expression of *BAX* and *TP53* in cells treated with resveratrol/DOX and DID/DOX was found, along with negative regulation of the expression of the antiapoptotic gene *BCL-XL*, results which suggests that the combination of these treatments induces apoptosis [70]. Similarly, combined treatments with the terpene borneol and DOX increased the intracellular capture of DOX and, moreover, favored the overproduction of ROS on human glioma cancer models. This resulted in an activation of p53 and p21, which in turn induced cell cycle arrest in the G2/M phase and suppressed angiogenesis [71]. Likewise, studies using *in vitro* models of pancreatic cancer demonstrated that the combination of the alkaloid piperlongumine and DOX increases cell apoptosis through the positive-regulation of caspase 3 and PARP (Poly(ADP-Ribose) Polymerase) [72].

In another study, Wen et al. evaluated the effect of curcumin on chemoresistance to DOX in MCF-7/DOX andMDA-MB-231/DOX-resistant breast cancer cells. DOX is one of the most commonly-used chemotherapeutic agents in this type of cancer, but the long-term benefits of this treatment are limited by resistance. This study showed sensitization of the cells resistant to DOX treatment mediated by the inhibition of the ATPase activity of ABCB4 (an efflux carrier involved in the elimination of intracellular drugs), thereby improving the accumulation of the therapeutic agent in the cancer cell and thus the treatment response in resistant cells [72].

Vielanin K and Vielanin P are terpenoids derived from *Xylopia vielana* leaves. Combined treatments with vielanin K and DOX have increased sensitivity in DOX-resistant MCF-7 breast cancer cells through the activation of the IRE1α-TRAF2-JNK pathway, which in turn activates the intrinsic apoptotic pathway [73]. Moreover, at low cytotoxicity concentrations, vielanin P has been shown to increase intracellular DOX accumulation, reduce the formation of colonies, and increase DOX-induced apoptosis through the inhibition of the PI3K/Nrf2 signalling pathway, which affects the expression and function of the ATP-binding cassette transporter MRP1 in MCF-7 and K562 DOX-resistant cell lines [74].

DOX is also one of the most commonly-used drugs in the treatment of osteosarcoma, however, it is greatly limited due to the high cardiotoxicity rate it presents if used at high concentrations [57]. On the other hand, it has been demonstrated that its use at low doses not only reduces its effectivity but also leads to resistance to the drug [57], therefore, it is vital to found alternative strategies to increase the survival rate of osteosarcoma patients [75]. Long non-coding RNAs (lncRNAs) has been shown to have important biological functions in tumor progression; a good example are the lncRNA transcript variants generated by human SOX2 overlapping transcript gene (SOXOT), which has been correlated with cellular differentiation and carcinogenesis [76]. Wang et al. observed that combined treatment of EGCG with DOX reduces the expression of the lncRNA SOXOT variant 7 on osteosarcoma cell models, improving the growth inhibition potential of DOX [77].

Sarvestani et al. evaluated the effect of combining DOX with two flavonoids, eupatorine and salvigenin, in colon cancer HT29 and SW948 cells and the normal fibroblastic cell line HFFF-2. The researchers found a synergistic effect between the two flavonoids used in combination with DOX, to induce reduced cell viability and the induction of ROS production and mitochondrial dysfunction, which results in apoptosis via the mitochondrial pathway [78],

Likewise, synergistic effects have also been reported for DOX in combination with alkaloids and terpenes [72, 79].

### 2.4 Paclitaxel

PTX is one of the most-commonly used taxane chemotherapeutics in the treatment of late-stage cancers, such as advanced prostate cancer, due to its potential to trigger apoptosis [80]. Naringin (4′, 5,7- 7-trihydroxyflavanone-aminoglucoside), is a natural glucoside known as a bioflavonoid, which is derived from grapefruit and other citrus fruits, which has been shown to posses anti-inflammatory, antioxidant and anticancer activities [81, 82, 83, 84]. Erdogan et al. evaluated the effect of the combination of PTX and naringin on prostate cancer cell lines and the results showed a synergistic effect of the compounds on the inhibition of cell migration capacity and on the increase in the expression of the tumor-suppressor protein PTEN (*Phosphatase and tensin homologue eliminated in chromosome 10*) [85].

Coralyne is a heterocyclic analogue of the protoberberine alkaloid that has antileukemic activity. In one study, combined treatments of coralyne and PTX showed a greater reduction in the expression of the proliferation-related protein ki-67, lower interaction of breast cancer cells with the extracellular matrix, an increase in Bax expression and a reduction of Bcl, when compared with the use of these compounds separately. This indicates strong synergistic activity in the inhibition of proliferation and migration, and the induction of the intrinsic apoptotic pathway in MCF-7 and MDA-MB-231 breast cancer cell lines [86].

Nuciferine is an alkaloid derived from the plants *Nelumbo nucifera* and *Nymphaea caerulea*. *In vitro* and *in vivo* experiments have shown that nuciferine can overcome drug resistance to PTX and other chemotherapeutics by the modulation of PI3K/AKT/ERK pathways, which results in the suppression of Nrf2 and HIF-1α activation, as well as their downstream targets, P-gp and BCRP (Breast Cancer Resistance Protein) in PTX-resistant cell lines from colon and lung cancer. This modulation induces the intracellular accumulation of chemoterapeutics agents, which finally induces cytotoxicity and tumor growth suppression [87].

Other alkaloids also show potential to improve the effects of PTX in chemotherapy. Studies suggest that piperine and piperlongumine increase the levels of intracellular ROS in SKOV-3 ovarian cancer cells and in INT-407 and HCT-116 intestinal cancer cells, which results in an increase in intrinsic apoptosis [88, 89].

### 2.5 Other drugs

Imatinib is a monoclonal antibody with inhibitory activity towards various quinase proteins, including those associated with the cluster region of the point of rupture in the ABL (Abelson) and BCR gene (Breakpoint Cluster Region), known as Bcr-Abl gene, the receptors of the growth factor derived from platelets (PDGF-R) and c-kit [90]. Additionally, it has been shown that the pleiotropic nature of curcumin enables it to strengthen the efficacy of imatinib and help reduce resistance to the drug in acute lymphoblastic leukaemia by the modulation of the AKT/mTOR pathway and the reduction of BCR/ABL gene expression, which results in apoptosis induction and inhibition of proliferation [91]. In a case report, Demiray et al., evaluated the intravenous and oral use of curcumin in combination with the chemotherapeutic imatinib in a 43-year-old patient diagnosed with adenoid cystic carcinoma of the submandibular saliva duct, with pulmonary metastasis, prior resistance to chemotherapeutic treatment (cisplatin + etoposide) and positive expression of c–kit in the tumor cells. The patient was treated with one dose of 400 mg/day of imatinib, in combination with 225 mg/of curcumin delivered intravenously twice per week and 2 × 84 mg/day of the oral bioavailable curcumin Arantal®. This resulted in a significant anatomical reduction of the tumoral mass without invasion of the mediastinal lymph nodes after 24 days of treatment and, after 6 months, an almost complete anatomical and metabolic improvement were observed, based on physical examination and normal laboratory evaluations. The researchers did not report secondary effects during the treatment period, taking into account that the intravenous curcumin was well-tolerated and no toxic effects or adverse reactions were observed [92].

Docetaxel, like PTX, is a second-generation chemotherapeutic drug from the taxane family, which is used in the treatment of different types of cancer. At molecular level, it bonds with high affinity to β-tubulin, altering the dynamic of the microtubules and affecting the functions of the cytoskeleton during mitosis. As a result, it affects the cell cycle, resulting in the arrest of the G2/M phase, which in turn leads to inhibition of proliferation and cell death [93]. In one study, the combination of piperlongumine and docetaxel showed strong anticancer activity due to the suppression of various proliferative and apoptotic molecular markers associated with breast cancer, and the negative regulation of Bcl-2 and survivin. Survivin is expressed in many types of cancer and is believed to perform important role in resistance to chemotherapy, thereby increasing tumoral recurrence and shortening patient survival [94].

One of the most beneficial chemotherapy agents in the treatment of glioblastoma and astrocytoma is temozolomide, an alkylating drug that causes the methylation of guanine nucleotides in DNA [95]. Wen-Jian Liu et al. demonstrated that borneol has the potential to improve the anticancer efficacy of temozolomide in human glioma U251 cells, through the induction of ROS production and positive regulation of apoptosis by mitochondria-dependent mechanism, which results in the activation of the caspases 3, 7 and 9, the proapoptotic proteins Bax and Bad and, the negative regulation of the antiapoptotic proteins Bcl-2 and Bcl-XL [96].

Gemcitabine is a standard chemotherapy agent used to treat advanced cancer of the pancreas, however, it was observed that patients rapidly develop resistance to gemcitabine [97]. A study carried out by Lou et al. suggests that the terpene ginkgolide B could improve sensitivity to gemcitabine in pancreatic cancer cell lines, through the modulation of cell proliferation, apoptosis and tumor growth in a xenograft tumor model with Capan-1 cells [98].

The chemotherapeutic agent oxaliplatin is widely used in the treatment of different types of gastrointestinal cancer. There have been recent studies of synergistic effects between this agent and polyphenolic compounds such as metabolites of ellagitaninns, lignans and isoflavones. Nordeny et al. shown that the combination of oxaliplatin with the ellagitaninn metabolite urolithin A, modulates the proliferative potential of HCT116 colon cancer cells by p53-dependent mechanism, which results in the induction of p21 and TIGAR (TP53-Induced Glycolysis And Apoptosis Regulator) expression. This is important, taking into account the modulation of cell cycle progression by p21 and the inhibition of the glycolytic potential of cancer cells by TIGAR, mechanisms that results in the limitation of cell growth [99].

### 2.6 Synergistic or additive effect?

Among the benefits of the use of combination treatments are the synergism that they can cause and their possible favorable results, which include increasing the efficacy of the therapeutic effect, decreasing the dose but increasing or maintaining the same efficacy to avoid toxicity and minimizing the development of drug resistance [100]. Because of these therapeutic benefits, drug combinations have been widely used and have become the main option for the treatment of diseases. In the context of this review, it was possible to determine that most of these studies show synergistic behavior, and only in specific cases additive, as shown in Table 2.

**Table 2:** Overview over the effect of the combined treatments of naturals compounds with chemotherapeutic agents. These drugs interaction was determined by the combination index (CI), coefficient of drug and ∗∗Q value.

Drug combinations	Doses (for this effect)	Effect			References
		Synergic	Additive	Value	
Curcumin + 5-Fluorouracil	5 μM of Curcumin + 0.01 nM of 5-Fluorouracil	x		-	[6]
Curcumin + 5-Fluorouracil	2.5–30 μM of Curcumin + 10–30 μM of 5-Fluorouracil	x		CI: 0.396	[12]
Curcumin + 5-Fluorouracil	>2.05 μM/L of Curcumin + >4.09 μM/L of 5-Fluorouracil	x		CI:0.2	[13]
Curcumin + Cisplatin	10 μM of Curcumin +10 ​μM of Cisplatin	x		-	[53]
Resveratrol + 5-Fluorouracil	5 μM of Resveratrol + 1nM of 5-Fluorouracil	x		-	[7]
Resveratrol + 5-Fluorouracil	25 μM of Resveratrol + 20 μM of 5-Fluorouracil	x		-	[26]
Resveratrol + Cisplatin	2.5 μM of Resveratrol+ 20 ​μM of Cisplatin	x		-	[39]
Resveratrol + Cisplatin	12.5 ug/ml of Resveratrol + 0.625 μg/ml of Cisplatin	x		CDI∗ < 1	[54]
Resveratrol + Doxorubicin	17.5 μM of Resveratrol + 0.52 μM of Doxorubicin 105 μM of Didox + 0.06 μM of Doxorubicin	x	x	Resveratrol CI: 1.02 Didox CI 0.6	[70]
Epigallocatechin-3-gallate + Cisplatin	20 μM of Epigallocatechin-3-gallate +10 ​μM of Cisplatin	x		CI: 0.72	[65]
Epigallocatechin-3-gallate + DOX	20 μg/ml of Epigallocatechin-3-gallate + 2.5 μM of Doxorubicin	x		CI: 0.794 ± 0.035	[77]
Urolithin A + Oxaliplatin	2.5 μM of Urolithin A + 0.85 ± 0.1 μM of Oxaliplatin	x		CI: 0.66	[99]
DPP-23 + Cisplatin	NR	x		CI: 0.61	[40]
Luteolin + Cisplatin	100 ​μM of Luteolin +2 μg/ml of Cisplatin	x		Q∗∗ = 1.22 ± 0.04	[55]
Caffeic acid + Cisplatin	10 μM of caffeic acid +5 μM of Cisplatin	x		-	[41]
Emetine + Cisplatin	Molar ratio 1:0.04	x		CI: 0.53	[49]
Neferine + Cisplatin	10 μM of Neferine + 10 μM of Cisplatin		X	CI: 1	[38]
Neferine + Cisplatin	6 μM ofNeferine + 15 μM of Cisplatin		x	CI: 1	[37]
Tetrandrine + Cisplatin	8 μM of Tetrandrine + 10 μM of Cisplatin	X		CI: 0.9	[50]
Piperlongumine + Docetaxel	MDA-MB-231: 1.2 μM of Piperlongumine + 0.12 μM of Docetaxel HS578T: 0.039 μM of Piperlongumine + 0.12 μM of Docetaxel MDA-MB-468: 0.0046 μM of Piperlongumine + 0.00046 μM of Docetaxel HCC1806: 0.0029 μM of Piperlongumine + 0.00029 μM of Docetaxel HCC70: 0.01074 μM of Piperlongumine + 0.001074 μM of Docetaxel0.001074	X		CI: 0.57 CI: 0.6 CI: 0.0022 CI: 0.16 CI: 0.058	[94]
Piperlongumine + Doxorubicin	1.0 μM of Piperlongumine + 0.05 μM of Doxorubicin	X		CI < 0.3	[72]
Coralyne + Paclitaxel	MCF-7: 15 μM of Coralyne+ 0.015 μM of Paclitaxel MDA-MB-231: 15 μM of Coralyne+ 0.015 μM of Paclitaxel	X		CI: 0.868 CI: 0.843	[86]
Nuciferine + Paclitaxel	3.79 μM of Nuciferine + 0.16 μM of Paclitaxel	X		CI: 0.064	[87]
Piperine + Paclitaxel	10 ​μM of Piperine + 5 ​μM of Paclitaxel	X		CI: 0.8036	[88]
Piperlongumine + Paclitaxel	INT-407: 5.0 μM of Piperlongumine + 1.0 μM of Paclitaxel HCT-116: 5.0 μM of Piperlongumine+ 1.0 μM of Paclitaxel	X		CI: 0.2469 CI: 0.2469	[89]
Oridonin + Cisplatin	20 μM of Oridonin+ 20 μM of Cisplatin	X		CI: 0.699	[36]
β-caryophyllene oxide and trans-nerolidol + Doxorubicin	5-500 ​μM of β-caryophyllene oxide+ 0.1-3 μM of Doxorubicin 5–500 ​μM of Trans-nerolidol+ 0.1 - 3 ​μM of Doxorubicin	X		CI: 0.2 CI: 0.7	[79]

The formula for $CI=\frac{a}{A}+\frac{b}{B}$, with a, b being the concentrations in the combination and A, B the singles doses of the compounds to medicate a given effect (Chou-Talalay method) and isobologram principles. CI values > 1 was indicative for antagonistic, CI = 1 for additive and CI values < 1 for synergistic action. ∗Coefficient of drug (CDI) value was calculated by the formula $CDI=\frac{AB}{\left(A\times B\right)}$, where AB represents the cell viability after cells incubated with a single compound alone. CDI <1 represents synergy of A and B, CDI = 1 represents additivity of A and B, and CDI >1 represents antagonism of A and B. ∗∗Q values were used in the Zheng-Jun Jin method for analyzed the inhibition rate between the combination of two compounds. The formula for the Q value is Q = Ea + b/(Ea + Eb - Ea × Eb), were Ea + Eb, Ea, and Eb are the inhibition rate of the combination group, drug a and drug b, respectively. Q = 1 would mean simple addition; Q > 1, synergistic or potentiation, Q < 1, antagonism.

### 2.7 Clinical assays

In addition to the effects observed both *in vitro* and in *vivo*, the outcomes of the combination of natural products and chemotherapeutic agents have been evaluated in some clinical trials, being curcumin, the most widely natural product used for the interventions. However, it is important to note that the low oral bioavailability of curcumin has been of great concern [101]. For this reason, different strategies have been explored *in vitro* and *in vivo* to overcome this drawback, such us complexed/encapsulated curcumin, curcumin formulations, curcumin nanoparticles and the use of adjuvants that interferes with curcumin metabolism [102].

In a phase II, single arm, single center trial it was evaluated the safety and efficacy of a oral formulation of curcumin in complexed form with phospholipids, as nutritional complement (2000 mg/day) to gemcitabine treatment administered to 44 patients with locally advanced or metastatic pancreatic cancer [103]. The results of the intervention shown absence of neurotoxicity and low hematological toxicity; likewise, it was observed a disease control rate of 61.4%, with a median progression free survival of 8.4 months and an overall survival (OS) of 10.2 months, which is higher to the OS of 6.7 months when using gemcitabine as single agent [104] and similar to the OS of 8.5–10.7 months when a combination of nanoparticle albumin-bound paclitaxel and gemcitabine were used [105].

Likewise, in a phase IIa open-labelled randomized controlled trial it was evaluated the safety and efficacy of folinic acid/5-fluorouracil/oxaliplatin chemotherapy (FOLFOX) compared with FOLFOX + 2 ​g of oral curcumin (Curcumin C3 Complex/d, containing ∼80% curcumin and 20% demethoxycurcumin and bisdemethoxycurcumin) named the CUFOX group, in 28 metastatic colorectal cancer patients. The results shown that the addition of daily curcumin to FOLFOX chemotherapy is safe, taking into account that the incidence rate of adverse events and the Global health scores were similar for both arms; likewise, higher and significant OS were observed in the CUFOX intervention group. Interestingly, they also analyzed plasma curcuminoid concentrations in order to assess protocol compliance and were able to detect curcumin, demethoxycurcumin and curcumin metabolites, like curcumin glucuronide and curcumin sulfate, in the CUFOX group [106].

Saghatelyan et al., performed a comparative, randomized, double-blind, placebo-controlled trial to evaluated the safety and efficacy of a intravenous curcumin infusion (300 mg solution, once per week) in combination with PTX in 150 patients with metastatic and advanced breast cancer. The results of the intervention shown that intravenous curcumin not led to reduction in quality of life and in terms of efficacy, curcumin group exhibited better objective response rates (defined as the complete response or partial response confirmed 4 weeks from the first response) than the placebo group, even 3 months after termination of the treatment [107]. Unfortunately, they do not analyze plasma curcuminoid concentrations to compare the benefits in terms of bioavailability, between oral and intravenous administration.

## 3 Conclusions

**Figure 3:**
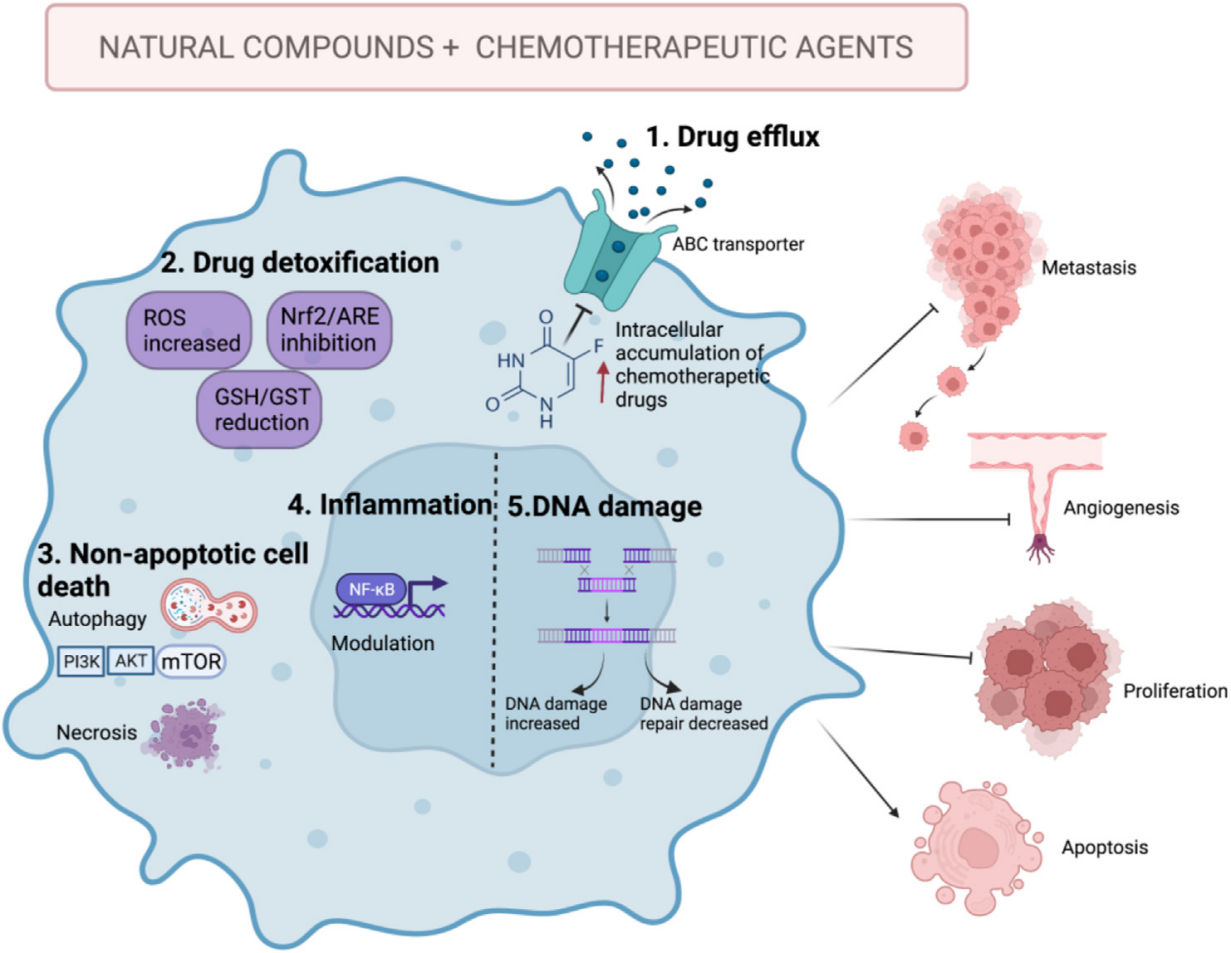
Mechanisms of action of the combined therapy of natural compounds and chemotherapeutic agents. Combination therapy presents an alternative to the therapeutic difficulties of cancer. (1) Inhibition of efflux pumps, induces the intracellular accumulation the chemotherapeutic agent; (2) Tumor cells increase the expression of genes related to the detoxification of xenobiotics; combined therapy provides inhibition of these mechanisms accompanied by an increase in the production of ROS. (3) Non-apoptotic cell death such as autophagy and necrosis are activated by combination therapy. (4) Proinflammatory cytokines act mainly due to the activation of the transcription factor NF-κB. This transcription factor can induce the expression of various genes that modulate apoptosis and promote cellular transformation, progression and chemoresistance; natural compounds with immunomodulatory activity have been reported as possible adjuvants to chemotherapy. (5) Resistance to chemotherapeutics can be generated by increased ability of cancer cells to repair DNA damage, thereby considerably reducing the efficacy of the drug. Inhibit the activity of the repair mechanisms and enhanced DNA damage increased cell death and reduced proliferation. These mechanisms result in decreased cell proliferation and metastatic capacity, reduced angiogenesis and in some cases increased apoptosis. Created with BioRender.com ABC: ATP binding cassette, ROS: reactive oxygen species.

Significant advances have been made in the development of strategies for the management of cancer patients, however, it is still observed that clinical response to cancer-therapy is limited, which has promoted the search for alternatives to improve its efficacy. In this regard, natural compounds have demostrated to posses a wide range of pharmacological effects and also to be effective in the improvement to drug resistance in cancer therapy. As we shown in this literature review, the mechanisms presented by natural compounds in enhancing chemotherapy are: (1) Inhibition of efflux pumps, which allows higher intracellular concentrations of the chemotherapeutic drugs, (2) Induction of ROS production and modulation of gene expression and activity of proteins involved in xenobiotic detoxification, (3) Activation of mechanisms of non-apoptotic cell death, such as autophagy and necrosis, (4) Modulation of proinflammatory signalling pathways, (5) Inhibition of repair mechanisms, to enhance the genotoxic potential of chemoterapeutic drugs (Figure 3).

These observations are of great relevance, considering that this mechanism includes categories related with several “hallmarks” capabilities of cancer cells, which are involved in the modulation of proliferation, angiogenesis, migration and invasion. Given the available knowledge on the effect of natural products on tumor cells, and their specific actions in enhancing chemotherapy, their concomitant future use with chemotherapeutics in the combined treatment of different types of cancer can be considered. However, the toxicity of the interactions between these natural compounds and chemotherapeutic drugs on the hepatic and renal tissue must be carefully considered.

This evidence demonstrates the need for clinical trials that allow the analysis of the synergistic and additive effects found on *in vitro* models, which allow the improvement of the combination treatment and its possible inclusion in the clinical practice.

## Declarations

### Author contribution statement

All authors listed have significantly contributed to the development and the writing of this article. </p>

### Funding statement

This work was supported by the Instituto Tecnológico Metropolitano and by a Minciencias grant (project code: 115080763215 CT 811–2018).

### Data availability statement

Data included in article/supp. material/referenced in article.

### Declaration of interest’s statement

The authors declare no conflict of interest.

### Additional information

No additional information is available for this paper.
